# Paroxysmal Dystonic Posturing Mimicking Nocturnal Leg Cramps as a Presenting Sign in an Infant with DCC Mutation, Callosal Agenesis and Mirror Movements

**DOI:** 10.3390/jcm13041109

**Published:** 2024-02-16

**Authors:** Adriana Prato, Lara Cirnigliaro, Federica Maugeri, Antonina Luca, Loretta Giuliano, Giuseppina Vitiello, Edoardo Errichiello, Enza Maria Valente, Ennio Del Giudice, Giovanni Mostile, Renata Rizzo, Rita Barone

**Affiliations:** 1 Child Neurology and Psychiatry Unit, Department of Clinical and Experimental Medicine, University of Catania, 95123 Catania, Italy; adrianaprato01@gmail.com (A.P.); lara.cirnigliaro@phd.unict.it (L.C.); fede-maugeri@hotmail.it (F.M.); rerizzo@unict.it (R.R.); 2 Department “G.F. Ingrassia”, Section of Neurosciences, University of Catania, 95123 Catania, Italy; antolucaster@gmail.com (A.L.); giuliano.loretta@gmail.com (L.G.); g.mostile@unict.it (G.M.); 3 Department of Molecular Medicine and Medical Biotechnologies, University of Naples Federico II, 80131 Naples, Italy; giuseppina.vitiello@unina.it; 4 Department of Molecular Medicine, University of Pavia, 27100 Pavia, Italy; edoardo.errichiello@unipv.it (E.E.); enzamaria.valente@unipv.it (E.M.V.); 5 Neurogenetics Research Center, IRCCS Mondino Foundation, 27100 Pavia, Italy; 6 Child Neurology, Department of Translational Medical Sciences, University of Naples Federico II, 80131 Naples, Italy; ennio.delgiudice@unina.it; 7 Research Unit of Rare Diseases and Neurodevelopmental Disorders, OASI Research Institute-IRCCS, 94018 Troina, Italy

**Keywords:** DCC, mirror movements, ACC, dystonic postures

## Abstract

**Background/Objectives**: Pathogenic variants in the deleted in colorectal cancer gene (DCC), encoding the Netrin-1 receptor, may lead to mirror movements (MMs) associated with agenesis/dysgenesis of the corpus callosum (ACC) and cognitive and/or neuropsychiatric issues. The clinical phenotype is related to the biological function of DCC in the corpus callosum and corticospinal tract development as Netrin-1 is implicated in the guidance of developing axons toward the midline. We report on a child with a novel inherited, monoallelic, pathogenic variant in the DCC gene. **Methods**: Standardized measures and clinical scales were used to assess psychomotor development, communication and social skills, emotional and behavioural difficulties. MMs were measured via the Woods and Teuber classification. Exome sequencing was performed on affected and healthy family members. **Results**: The patient’s clinical presentation during infancy consisted of paroxysmal dystonic posturing when asleep, mimicking nocturnal leg cramps. A brain magnetic resonance imaging (MRI) showed complete ACC. He developed typical upper limb MMs during childhood and a progressively evolving neuro-phenotype with global development delay and behavioural problems. We found an intrafamilial clinical variability associated with DCC mutations: the proband’s father and uncle shared the same DCC variant, with a milder clinical phenotype. The atypical early clinical presentation of the present patient expands the clinical spectrum associated with DCC variants, especially those in the paediatric age. **Conclusions**: This study underlines the importance of in-depth genetic investigations in young children with ACC and highlights the need for further detailed analyses of early motor symptoms in infants with DCC mutations.

## 1. Introduction

Agenesis of the corpus callosum (ACC) is one of the most common congenital brain malformations consisting of the complete or partial absence of the corpus callosum and affecting approximately 1 in 4.000 newborns [1,2]. Malformations in the corpus callosum are observed in various conditions that disrupt early cerebral development occurring from week 13 to 20 post-conception, and can be frequently associated with other cerebral or extracranial malformations [3]. Pathogenic variants in single genes and complex genetics have been involved in the aetiology of ACC [4].

The deleted in colorectal carcinoma (DCC) gene (MIM *120470) located in chromosome region 18q21.2 encodes the receptor for netrin-1 (NTN1) which promotes neuronal growth in the developing nervous system [5]. The originally described function of the DCC gene as an oncosuppressor [6] has not been demonstrated in vivo and remains controversial [7]. However, somatic variants in the DCC have been reported in sporadic colorectal and oesophageal cancer [8]. NTN1 is implicated in the guidance of developing axons toward the midline, a crucial step in corpus callosum development [9]. Mice with homozygous null DCC mutations have severe defects in commissural development in the brain and spinal cord, with an absent corpus callosum and a decreased number and misrouting of commissural axons [10,11]. Monoallelic and biallelic DCC pathogenic variants have previously been associated with ACC [5,7]. Biallelic loss-of-function DCC variants have also been reported in patients with developmental split-brain syndrome (DSBS, MIM# 617542), a more complex syndrome associated with ACC, intellectual disability, scoliosis and horizontal gaze palsy, with or without mirror movements (MMs) [12]. Individuals with DSBS have a poor developmental outcome compared to individuals with DCC-ACC, likely attributed to additional brain abnormalities affecting the formation of other commissural tracts [5].

Heterozygous pathogenic variants in the DCC are also associated with congenital MMs. These are involuntary contralateral movements in one side of the body that accompany and mirror intentional movements on the opposite side, mostly involving the distal upper limbs [13,14]. MMs related to DCC variants usually present during infancy or early childhood and persist through adulthood with no overt aggravation [7,8].

Patients with ACC and DCC variants may also present with a wide spectrum of neurobehavioural features, including cognitive or developmental delay, impairments in emotional and social functioning, attention or visuospatial deficits, language disorders [5,15].

We report on a patient harbouring a novel pathogenic variant in the DCC gene, shared by all affected individuals in his family. The proband’s atypical clinical presentation during infancy consisted of paroxysmal episodes characterized by dystonic posturing when asleep, mimicking recurring nocturnal leg cramps, that necessitated clinical investigations. Upon examination, complete ACC was identified. He developed typical upper limb MMs during childhood and a progressively evolving neuro-phenotype illustrating the clinical spectrum of DCC mutations in paediatric patients.

## 2. Materials and Methods

### 2.1. Ethics Consideration

This study was based solely on information and investigations that were carried out as part of the routine clinical care of patients with neurological diseases. All procedures performed were in accordance with the ethical standards of the local Ethical Committee and with the 1964 Helsinki declaration and its later amendments. Written informed consent was signed by the parents of the proband.

### 2.2. Standardized Measures and Clinical Scales

Psychomotor development and cognitive level were assessed according with age by measuring the general quotient (GQ) through the Griffiths Mental Development Scales (GMDS) [16,17] (Hogrefe, Oxford, UK) and by using the intelligence quotient (IQ) on the Wechsler Intelligence Scale for Children—Fourth Edition (WISC-IV) [18] (Pearson, San Antonio, TX, USA) respectively. The Social Communication Questionnaire (SCQ) was used to evaluate communication skills and social functioning. The total score was interpreted with reference to a cut off score of 15 [19] (Western Psychological Services, Los Angeles, CA, USA). The Child Behaviour Checklist (CBCL) (ASEBA, Burlington, VT, USA) and the Conners’ Parent Rating Scale-Revised short form (CPRS-R:S) (MHS, New York, NY, USA) were used to assess emotional and behavioural difficulties, and inattention and hyperactivity problems, respectively. The suggested cut-offs provided by the manuals (T scores ≥ 65) were considered [20]. MMs were measured via the Woods and Teuber classification [21]. This classification scores MMs at three upper limb sites (fingers, wrists and forearms) from 0 (no clearly imitative movement) to 4 (movement equal to that expected for the intended hand) based on the observation of MMs in the contralateral limb. Total scores for the right (R) and left (L) sides were calculated by adding the individual 0–4 scores for finger flexion/extension, wrist flexion/extension and forearm pronation/supination.

### 2.3. Exome Sequencing (ES)

ES was performed on blood DNA samples of individuals III-2, II-2, II-3 and II-6 using the SureSelect Human All Exon v6 kit (Agilent Technologies, Santa Clara, CA, USA) on a HiSeq 2500 platform (Illumina, San Diego, CA, USA). The bioinformatic analysis was focused on heterozygous variants shared by all affected individuals (III-2, II-3, II-6) with frequency <1% in gnomAD and our in-house database, using a virtual panel of genes associated with ACC in HPO (HP:0001274). Candidate variants were classified according to the ACMG/AMP guidelines [22]. Sanger sequencing was used for variant validation and further segregation analysis was carried out on other available family members (I-3, II-4, II-5).

## 3. Results

### 3.1. Clinical Report

**Figure 1:**
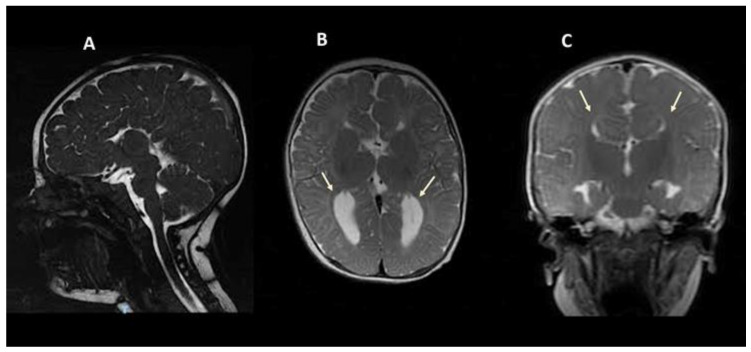
Brain magnetic resonance imaging (MRI) of the proband at age 6 months. Midsagittal FIESTA sequence (**A**) and axial and coronal T2-weighted sequences (**B**,**C**) show complete corpus callosum agenesis (ACC) with associated colpocephaly (**B**) and Viking helmet appearance of the lateral ventricles (**C**).

The child is a 7-year-old male of unrelated Italian parents. He was delivered vaginally preterm at 35 weeks and 6 days of gestation following a pregnancy complicated by threats of preterm birth during the third trimester. At-birth weight was 3010 g (50–90th pc), length was 48 cm (50–90th pc) and head circumference was 32 cm (10–50th pc). Apgar scores were 1′ 9, 5′ 10. Due to brain abnormalities found on the previous foetal ultrasound survey, a neonatal brain ultrasound (US) was performed with evidence of enlargement of the lateral ventricles and suspected ACC. Routine laboratory analyses and extended newborn metabolic screening yielded normal results. A brain magnetic resonance imaging (MRI) at the age of 6 months showed complete ACC with disproportionate enlargement of the occipital horns of the lateral ventricles (Figure 1). 

Psychomotor development was delayed: he sat unsupported at 9 months and gained independent walking at 20 months. He pronounced his first words at 24 months, with a subsequent delay in language development.

From the age of 6 months, he suffered from recurrent episodes of dystonic posturing of the lower limbs when asleep, mimicking nocturnal leg cramps, sometimes associated with crying. These episodes occurred one to five times per week with a mean duration of about 2 min. They were mostly unilateral involving the ipsilateral foot and sometimes bilateral (Video S1).

Following these clinical manifestations, he first came to our attention at the age of 18 months. Craniofacial dysmorphism was noted with a triangular face, high hairline, high forehead and wide nasal bridge. On neurological examination he had alternating strabismus, hypertonia of the lower limbs with hyperreflexia and bilateral foot clonus. The gait was unstable with a wide base. Electroencephalogram (EEG), ophthalmic and orthoptic examinations were normal. At the age of 3 years, MMs became evident in the upper limbs with sustained repetitive movements contralateral to the intended hand. While squeezing and releasing a toy by handling it with rotational wrist movements, he showed contralateral movements with a similar intensity of flexion, hand relaxation and wrist rotation. Repeated sleep EEG studies and routine blood investigations, including creatine kinase levels, were normal.

At the age of 30 months, psychometric evaluation using the Griffiths Mental Development Scales (GMDS) showed a global psychomotor development delay (general development quotient: 73), with lower scores on items evaluating performances and eye and hand coordination. At a subsequent evaluation using the Griffiths III (age 4 years), lower scores were found on items assessing memory skills, attention, reasoning, planning solutions and on items evaluating balance, such as motor planning, visuo-motor and overall body coordination (development general score: 50).

At the age of 6 years, formal assessment by the Wechsler Intelligence scale for Children—Fourth Edition (WISC-IV) indicated moderate intellectual disability (full-IQ: 49). The Child Behaviour Checklist (CBCL) and the Conners Parent Rating Scale (CPRS) were administered to the parents and revealed normal scores, except for the subscale “Cognitive/Attention problems”. The Social Communication Questionnaire (SCQ) was consistent with the presence of mildly impaired social skill (total score: 17). 

At last follow-up visit (age 7) neurological examination showed generalized hypotonia with hypertonia of the lower limbs, decreased muscle strength in the upper and lower limbs, hyperreflexia and mild Achilles tendon retraction. General clumsiness, MMs with impaired fine bimanual activities and stereotyped movements of the mouth and hands were observed. MMs were bilateral and symmetric with total Woods and Teuber scores of nine in both the right and left upper limbs (Video S2).

Furthermore, restlessness and behavioural problems with easy irritability, temper and crying tantrums were reported. Nocturnal paroxysmal dystonic posturing of the lower limbs persisted.

### 3.2. Family Background

The phenotypic study of the family was performed after the identification and the characterization of the index case (III-2). The father (II-3, aged 37 years) was affected by juvenile myoclonic epilepsy following pharmacological treatment. He had partial ACC on a brain MRI and showed bilateral MMs with a Woods and Teuber score of four in both the right and left upper limbs (Video S3). His cognitive level was below the normal range on the Wechsler Adult Intelligence Scale-R (total IQ: 68). 

The mother (II-2, aged 35 years) had no abnormalities on neurological examination.

A paternal uncle of the child (II-6, aged 34 years) displayed MMs of the hands (R3, L3), also reporting episodic abnormal posturing of the upper limbs. He showed no abnormalities on a brain MRI and his cognitive abilities were normal.

**Figure 2:**
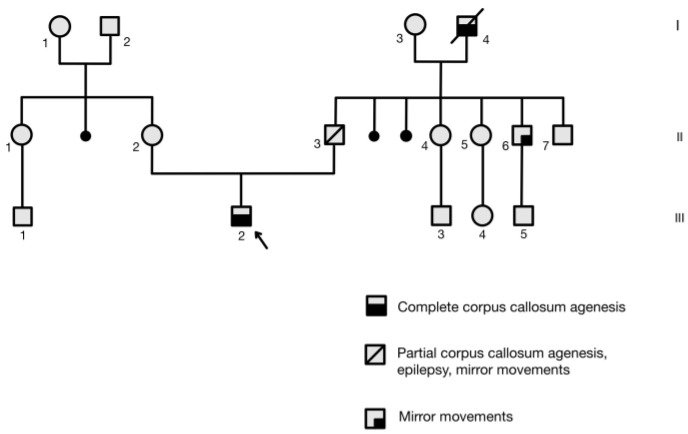
Neuroanatomic features and mirror movements (MMs) in family members with DCC mutation. I-4 and III-2: Complete ACC, MMs. II-3: Partial ACC, MMs. II-6: Normal corpus callosum (CC), MMs. Arrow indicates the proband. Numbers 1-7 indicate individual family members for each generation.

In addition, family history reported the presence of ACC and MMs in the paternal grandfather (I-4) (Figure 2).

### 3.3. Genetic Findings

ES detected a heterozygous missense variant in the exon 16 of the DCC [NM_005215.4:c.2426A>G, p.(Tyr809Cys)], shared by all affected individuals and absent in healthy family members, including the mother. No other gene variants were found in mother’s exome data. This change, which falls within the Fibronectin type-III 4 domain of the netrin receptor DCC (aa 728–821), is absent from population databases such as gnomAD, predicted as damaging by several in silico tools (e.g., CADD, EIGEN, PROVEAN, SIFT) and is classified as likely being pathogenic according to the ACMG/AMP guidelines.

## 4. Discussion

Heterozygous pathogenic variants in the DCC may lead to congenital MMs, associated with abnormalities of the corpus callosum and concomitant cognitive and/or neuropsychiatric issues. The present patient harboured a novel monoallelic DCC missense variant associated with delayed psychomotor development, intellectual disability, MMs and complete ACC. During infancy, he presented with an uncommon clinical manifestation characterized by nocturnal recurrent abnormal posturing of the legs mimicking cramps, leading to further medical investigations. Throughout the years, he experienced periodic occurrences of comparable episodes while sleeping.

Adult individuals with DCC-MMs can exhibit a range of functional disabilities besides difficulties in fine bimanual activities. These include fatigue, spontaneous muscle contractions and pain in the upper limbs during extended manual activities, such as writing, as well as general clumsiness and compensatory manoeuvres to inhibit involuntary movements [5,9,23,24].

At the time of writing, twenty-six children, including the present one, have been reported, harbouring 17 different DCC variants. However, only a subset of the children diagnosed with DCC-MMs were reported to have undergone brain imaging (12 out of 25; 48%) and formal neuropsychological evaluation (7 out of 25; 28%), including the studied patient (Table 1). Twenty-five (96%) had MMs, nine had partial or complete ACC (35%), eight had a combination of both (31%). Heterogeneous neurological features and neuropsychological performances were reported: six patients (23%) presented with development delay and low to borderline intellectual functioning, one patient was diagnosed with dyslexia and four (15%) had reading and language deficits combined with attention deficit disorder. One patient, diagnosed with autism spectrum disorder, presented with executive–attentive function deficits and seizures, another patient had seizures and chorea. Difficulties in fine bimanual activities and general clumsiness were described in about half of the cases. Six out of nine patients with partial or complete ACC (67%) also had other MRI brain abnormalities (colpocephaly, asymmetric ventriculomegaly, absence of cingulate gyrus). Muscle cramps have not been reported in children with DDC-MMs; these symptoms are often overlooked at a younger age, underlying the relevance of better assessing early neurological symptoms related to DCC mutations in childhood which could mimic cramps such as paroxysmal dyskinesia.

Previous studies showed the crucial role of the DCC/Netrin-1 system in axonal pathfinding implicated in the guidance of developing axons toward the midline. This is required for corpus callosum development and during corticospinal tract (CST) development. The corpus callosum is a white-matter bundle that acts as the main source of interhemispheric connectivity. Congenital MMs may occur in some individuals with corpus callosum dysgenesis indicating insufficient interhemispheric inhibition between the two primary motor cortices [29]. MMs have been recently linked to DCC mutation with or without ACC [9]. In children with unilateral and bilateral cerebral palsy (CP), stronger MMs were related to a decreased integrity of transcallosal fibres and were also associated with abnormal wiring of corticospinal tracts, suggesting that a lack of interhemispheric inhibition might be a possible mechanism underlying an increased MM-intensity [30,31,32]. Nocturnal leg cramps are rather uncommon in early childhood with an overall incidence of 7.3% between 3 and 18 years of age [33]. Nocturnal cramps in children manifest with sudden, involuntary and painful contractions usually involving the calf muscles accompanied by residual tenderness in the affected muscles and causing distress and sleep disruption. The pathophysiological mechanisms causing nocturnal leg cramps are diverse and encompass abnormal excitability and instability in the spinal anterior horn cells [34,35]. In our case, nocturnal paroxysmal posturing of the leg may be better explained by abnormal interhemispheric inhibition with unproper unilateral or bilateral CST wiring as observed in patients with MMs, ultimately leading to spinal anterior horn cells’ activation. Further studies focusing on evaluation of interhemispheric facilitation and inhibition paradigms are required to investigate to what extent MM and paroxysmal dyskinesia occurrence depends on the interhemispheric connectivity in DDC mutation disease.

According to previous studies [4,13,15,27], we found an intrafamilial clinical variability as to neurological and neuroanatomic features associated with DCC mutations. In particular, in addition to the proband, two more family members harboured the same missense DCC variant. The child’s father had MMs and partial ACC, mild intellectual disability, episodic dystonia and epilepsy while the affected paternal uncle had isolated MMs and episodic limb dystonia with no brain malformation and no intellectual impairments. Further research is necessary to comprehend the intricacy of the clinical phenotype linked to DCC variations, especially in cases of missense alterations [5,13], such as the one examined in this study. Possible phenotypic modifiers of DCC mutations may also be gender related. Actually, in individuals with truncating DCC variants, ACC is more often present in females; in individuals with missense variants, no significant gender differences in ACC prevalence are reported. Moreover, MMs were reported to be more common in males than in females. Sex-biased phenotypic expression of ACC and MMs in individuals with DCC mutations may be associated with the regulation of the DCC by testosterone levels during prenatal brain development [7]. Additional unknown genetic modifiers or other unidentified neurological or neuroanatomical factors may influence the phenotype thus explaining the observed inter and intrafamilial variability associated with DCC mutations. The neurophysiological and phenotypic heterogeneity associated with DCC mutations suggests the importance of an in-depth clinical and neuropsychological assessment in patients with ACC and MMs having a normal lifespan, over time [4,36,37]. In this regard, a recent study described five novel DCC variants in members of three families with CMM with age ranging from 6 to 67 years [13]. DCC mutations associated with MMs may preclude affected individuals from professions and social activities that demand sustained or complex bimanual coordination. In this study, the proband was intellectually impaired and had reduced performances in a wide range of cognitive domains. Behavioural concerns occurred at school age while personal–social–emotional skills worsened over time. Notably, in patients with DCC mutations, cognitive, behavioural and academic disturbances can emerge with age with a risk of increased severity when both ACC and MMs are present [15]. Therefore, a follow-up over time is required to monitor the clinical course of the disturbances in children with DCC mutation, providing tailored interventions according to child’s individual difficulties. 

## 5. Conclusions

The early clinical presentation of the present patient, consisting of nocturnal paroxysmal dyskinesia mimicking leg cramps, expands the clinical spectrum associated with DCC variants especially in the paediatric age. This study underlines the importance of in-depth genetic investigations in young children with ACC and highlights the need for further detailed analyses of early motor symptoms in infants with DCC mutations.

## Figures and Tables

**Table 1:** Clinical overview of monoallelic DCC variants in the paediatric-age patients.

Patient- Gender/Age	Ethnicity	Development	Cognition	MMs	Other Neurological Manifestations	CC	Other Brain Abnormalities	DCC Variant	Reference
M/10	Caucasian	Normal	Normal	+	Difficulties in fine bimanual activities	NA	NA	c.3835_3836delCT/p.Leu1279ProfsX24	Depienne et al., 2011 [9]
M/18	Turkish	NA	NA	+	Difficulties in fine bimanual activities	NA	NA	c.2000 G>A/p.Arg667His	Meneret et al., 2014 [25]
M/6	Caucasian	NA	NA	+	Difficulties in fine bimanual activities; general clumsiness	NA	NA	c.1336_1337insAGCC/p.Arg446GlnfsX27	Meneret et al., 2014 [25]
F/8	Caucasian	NA	NA	+	Difficulties in fine bimanual activities	NA	NA	c.2871_2875dup/p.Pro960GlyfsX8	Meneret et al., 2014 [25]
F/3	Caucasian	NA	NA	+	Difficulties in fine bimanual activities; general clumsiness	NA	NA	c.823C>T/p.Arg275X	Meneret et al., 2014 [25]
F/6	North African	NA	NA	+	Retinal dystrophy; difficulties in fine bimanual activities and writing; general clumsiness	NA	NA	c.823C>T/p.Arg275X	Meneret et al., 2014 [25]
F/11	North African	NA	NA	+	Retinal dystrophy; difficulties in fine bimanual activities; general clumsiness	cACC	-	c.823C>T/p.Arg275X	Meneret et al., 2014 [25]
M/7	Caucasian	NA	NA	+	Difficulties in fine bimanual activities and everyday tasks	NA	NA	c.377C>A/p.Ser126X	Meneret et al., 2014 [25]
F/6	Caucasian	Normal	Normal	+	Clumsiness; inability to perform unimanual or skilled dissociated movements of the two hands	NA	NA	c.823C>T/p.Arg275X	Meneret et al., 2015 [23]
M/6	Caucasian	Normal	Normal	+	Clumsiness; difficulties in fine bimanual activities	NA	NA	c.1999dupC/p.Arg667Profs*4	Bierhals et al., 2018 [13]
M/6	Caucasian	Motor delay	Normal	+	Clumsiness; difficulties in fine bimanual activities	NA	NA	c.1962delT/p.Phe654Leufs*46	Bierhals et al., 2018 [13]
M/11	Caucasian	Normal	Normal	+	Difficulties in fine bimanual activities, migraine	NA	NA	c.4211_4215dup/p.Ser1406Lysfs*22	Bierhals et al., 2018 [13]
F/13	Caucasian	Normal	NA	+	NA	NA	NA	c.4211_4215dup/p.Ser1406Lysfs*22	Bierhals et al., 2018 [13]
M/4	Ethiopian	Normal	Normal	+	-	Normal	-	c.2774dupA/Asn925Lysfs*17	Sagi-Dain et al., 2020 [26]
F/9	Iranian	Normal	NA	+	NA	Normal	-	c.1729delG/p.Glu577Argfs*12	Thams et al., 2020 [27]
F/4	Iranian	NA	NA	+	NA	NA	NA	c.1729delG/p.Glu577Argfs*12	Thams et al., 2020 [27]
M/9	Caucasian	NA	NA	+	Dyslexia	Normal	-	c.1466_1476del/p.Val489Glufs*15	Thams et al., 2020 [27]
M/7	Caucasian	Language delay	Normal	+	Language disorder; chorea; seizures	cACC	-	c.1466_1476del/p.Val489Glufs*15	Thams et al., 2020 [27]
F/11	North African	Language delay	Low average intellectual functioning	-	Reading difficulties; attention deficit	pACC	-	c.925delA/p.Thr309Profs*26	Marsh et al., 2017 [7]; Spencer-Smith et al., 2020 [15]
M/12	Caucasian	Motor and language delay	Low intellectual functioning	+	Global behavioural/academic impairments; ADHD	cACC	Colpocephaly; absence of cingulate gyrus and posterior commissure	c.2378T>G/p.Val793Gly	Marsh et al., 2017 [7]; Spencer-Smith et al., 2020 [15]
M/10	Caucasian	Language delay	Borderline intellectual functioning	+	Global behavioural/academic impairments; ADHD	cACC	Colpocephaly; absence of cingulate gyrus and posterior commissure	c.2378T>G/p.Val793Gly	Marsh et al., 2017 [7]; Spencer-Smith et al., 2020 [15]
M/8	Caucasian	Motor and language delay	Low intellectual functioning	+	Global behavioural/academic impairments; ADHD	cACC	Colpocephaly; absence of cingulate gyrus	c.2378T>G/p.Val793Gly	Marsh et al., 2017 [7]; Spencer-Smith et al., 2020 [15]
M/10	Caucasian	Motor and language delay	Low intellectual functioning	+	ASD; seizures; behaviour problems; executive-attentive functions deficits; impaired coordination and ability to complete bimanual tasks	pACC	Colpocephaly	c.2414G>A/p.Gly805Glu	Marsh et al., 2017 [7]; Spencer-Smith et al., 2020 [15]; Knight et al., 2023 [4]
F/12	Caucasian	Motor delay	Normal	+	Executive functioning problems; behaviour problems; high anxiety symptoms	Normal	-	c.2414G>A/p.Gly805Glu	Marsh et al., 2017 [7]; Spencer-Smith et al., 2020 [15]; Knight et al., 2023 [4]
F/2	Israelian	Mild gross and fine motor delay	Normal cognitive and communication skills	+	-	pACC	Asymmetric ventriculomegaly	c. 2T>C/p.Met1*	Nissenkorn et al., 2021 [28]
M/7	Caucasian	Global development delay	Low intellectual functioning	+	Alternating strabismus; language disorder; episodes of stiffness with hyperextension of the lower limbs	cACC	Colpocephaly	c.2426A>G/p.Tyr809Cys	This report

cACC: complete agenesis of the corpus callosum; pACC: partial agenesis of the corpus callosum; ID: intellectual disability; +: present;-absent; NA: not available.

## Data Availability

The original contributions presented in the study are included in the article/Supplementary Material, further inquiries can be directed to the corresponding author.

## Supplementary Materials

The following supporting information can be downloaded at: https://www.mdpi.com/article/10.3390/jcm13041109/s1, Video S1: Paroxysmal dystonic posturing of the child’s lower limbs (home-made video); Video S2: Mirror movements of the child’s upper limbs; Video S3: Mirror movements of the child’s father hands.
